# NHC Core Pincer Ligands Exhibiting Two Anionic Coordinating Extremities

**DOI:** 10.3390/molecules25092231

**Published:** 2020-05-09

**Authors:** Rachid Taakili, Yves Canac

**Affiliations:** LCC–CNRS, Université de Toulouse, CNRS, 31077 Toulouse, France; rachid.taakili@lcc-toulouse.fr

**Keywords:** carbon ligand, amide, negative charge, NHC, phosphonium ylide, oxide, pincer

## Abstract

The chemistry of NHC core pincer ligands of LX_2_ type bearing two pending arms, identical or not, whose coordinating center is anionic in nature, is here reviewed. In this family, the negative charge of the coordinating atoms can be brought either by a carbon atom via a phosphonium ylide (R_3_P^+^–CR_2_^−^) or by a heteroatom through amide (R_2_N^−^), oxide (RO^−^), or thio(seleno)oxide (RS^−^, RSe^−^) donor functionalities. Through selected examples, the synthetic methods, coordination properties, and applications of such tridentate systems are described. Particular emphasis is placed on the role of the donor ends in the chemical behavior of these species.

## 1. Introduction

**Scheme 1:**
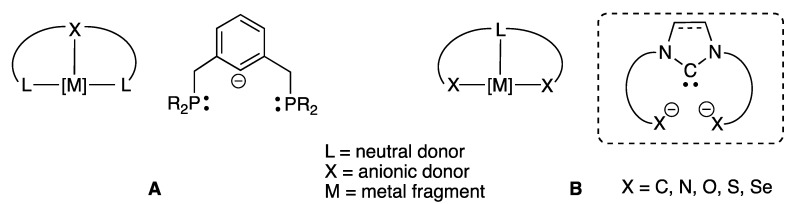
Representation of the XL_2_ prototype pincer **A** and LX_2_ pincers **B** of interest.

The term ‘pincer’ was introduced in 1989 by van Koten to refer to a tridentate ligand featuring a central anionic carbon atom coordinated through a covalent σ bond and associated with two flanking arms that donate their electron lone pairs and force the metal center to adopt a meridional geometry [1]. This geometry has the main advantage of leaving an open coordination shell for any incoming molecule and of preventing undesired ligand redistribution processes [2,3,4,5,6]. The high stability, variability and activity of pincer ligands have made these species essential today in modern organometallic chemistry and homogeneous catalysis [7,8,9]. The first pincer system prepared by Shaw et al. in the late 1970s was based on an anionic carbon atom belonging to a central phenyl ring and bearing two neutral pendant phosphine donors, the so-called PCP pincer ligands [10]. This pioneering report was followed shortly after by the preparation of the NCN analogues by van Koten and Noltes [11]. From there, a wide variety of chemical motifs were designed, and the term ‘pincer’ was generally extended to any metallic complex adopting a meridional geometry whatever the bonding mode of the coordinating ends, as opposed to the facial coordination mode found in tripodal systems [12,13,14]. According to the Green formalism [15], although mono-anionic pincers (L_2_X-type) remain the most exemplified, other systems containing neutral (L_3_-type), di- (LX_2_-type), or tri-anionic (X_3_-type) pincer frameworks were also developed allowing an efficient control of the metal coordination sphere and the stabilization of a broad range of metal centers with different oxidation states [16,17,18,19,20,21,22]. Among all pincer-type ligands, di-anionic representatives (LX_2_-type) have been relatively little explored, and considering only the cases where the central donor moiety is neutral [23], three main sub-structures of this family have been reported over the years featuring either a pyridine [24,25,26,27,28,29], a carbodiphosphorane [30,31,32], or a N-heterocyclic carbene (NHC) [33,34]. The latter architecture **B** built from a neutral NHC core (2-e^−^ donor (L-type)) and two anionic peripheral groups (1-e^−^ donor (X-type)) is formally characterized by an opposite bonding mode to that encountered in the Shaw’s prototype **A** derived from a central anionic aryl group (1-e^−^ donor (X-type)) and two neutral donor moieties (2-e^−^ donor (L-type)) (Scheme 1). In both structures, in addition to the thermodynamically favored chelate effect, the association of coordinating extremities of different nature extends naturally the scope of accessible metal fragments, but more generally of all centers having a Lewis acidic character. According to HSAB theory (hard soft acid base) [35,36], combining a soft NHC with two harder anionic groups must inevitably afford metallic complexes with unique properties, as is the case with systems **B**. For instance, a different bonding mode is anticipated with a high degree of covalency in the NHC-metal bond and a more marked ionic character between the negatively charged atoms and the metal center. It follows that anionic functionalized NHC ligands generally prefer to coordinate with electropositive metals, the anionic arms acting as a real anchor counterbalancing the natural tendency of the NHC to dissociate from the metal [37,38,39,40,41]. With late transition metals, an opposite behavior is expected with a possible lability of the anionic donor extremities [42,43,44]. All of these electronic criteria, combined with geometric parameters, govern the reactivity vs stability of these pincers, thus making each of them a unique system.

**Scheme 2:**
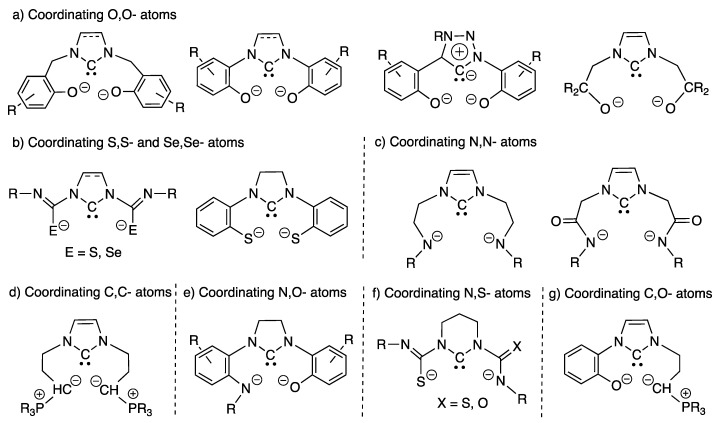
Representation of the general structure of known pincer structures of type **B** based on a central N-heterocyclic carbene (NHC) and two peripheral anionic coordinating atoms being identical or not.

This review aims to account for this class of LX_2_-type pincer ligands **B** where the central NHC is N-substituted by two side arms bearing identical or different anionic coordinating atoms, and which remains to this day underdeveloped compared to other families of pincer ligands. Through selected examples, the preparation methods, coordination properties, and applications of NHC core pincers exhibiting amide, oxide, thio(seleno)oxide, and phosphonium ylide donor extremities, which belong to this class of ligands, will be thus presented (Scheme 2).

## 2. NHC Core Pincer Ligands of LX_2_-Type

### 2.1. Based on Two Identical Side Arms

#### 2.1.1. With Two Coordinating O- Atoms

**Scheme 3:**
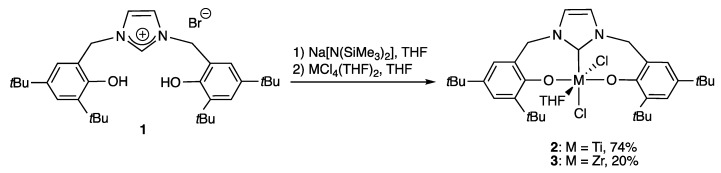
Preparation of group 4 pincer complexes **2**–**3** (M = Ti, Zr) featuring a bis(aryloxide) NHC ligand from the imidazolium salt **1** [45,46].

The first representatives of the family were reported by Kawaguchi et al. from the 1,3-bis(4,6-di-*tert*-butyl-2-hydroxybenzyl) imidazolium bromide **1** prepared in 60% yield via sequential N-alkylation of imidazole with 2-bromomethyl-4,6-di-*tert*-butylphenol. By reacting the imidazolium salt **1** with TiCl_4_(THF)_2_ in the presence of NaN(SiMe_3_)_2_, the bis(aryloxide) NHC Ti(IV) pincer complex **2** was formed in 74% yield (Scheme 3) [45]. An X-ray diffraction analysis confirms the meridional coordination mode of the NHC where the pseudo-octahedral geometry adopted by the Ti(IV) center is completed by a THF molecule and two chlorine atoms in mutual *cis* position. Using the same protocol, the Zr(IV) analogue **3** was prepared but with a lower yield (20%) since it was obtained as a mixture with the corresponding homoleptic bis(NHC) Zr complex isolated in 38% yield [46]. It should be mentioned that although the latter complex could be synthetized in 84% yield by the reaction of ZrCl_4_(THF)_2_ with two equivalents of ligand, attempts to prepare complex **3** in a better yield failed. The larger ionic radius of Zr with respect to Ti was proposed to explain the more favorable formation of the bis(NHC) complex in the case of Zr. Both pincer complexes **2** and **3** could be converted to their alkyl derivatives via chloride displacement upon addition of Grignard reagents. For example, the chloro ligands in complexes **2**–**3** could be substituted using 2 equivalents of PhCH_2_MgCl to form the corresponding dialkyl pincer complexes. Whatever the series, the benzyl complexes display a distorted trigonal bipyramidal geometry with a meridional coordination of the NHC.

Another striking fact concerns the dibromide analogue of complex **2**, which, upon reduction with an equivalent of LiBEt_3_H, led to the formation of a rare Ti(III) complex exhibiting a distorted octahedral geometry and where the coordination sphere around the metal is completed by the pincer ligand occupying meridional positions, two THF molecules in mutual *trans* position and a bromide co-ligand [47]. Following this, dinuclear complexes [48] and macrocyclic structures of the same tridentate ligand with group 4 metals were also reported by the same authors [49]. These metallic systems as well as the Ti(IV) pincer complex **2** showed significant catalytic activity for the polymerization of ethylene.

Based on the same flanking donor arms but with a central saturated six-membered NHC, rare earth pincer complexes (Nd, Y, Sm) were prepared, fully characterized, and applied in the polymerization of *n*-hexyl isocyanate [50]. The polymer formed showed very high molecular weight and narrow molecular weight distribution. It was also demonstrated that the catalytic activity depends on the radius of rare earth metal, solvent, polymerization temperature, and the ligand structure. The saturated NHC moiety was in particular found to play a critical role in the initiation step of the polymerization process.

**Scheme 4:**
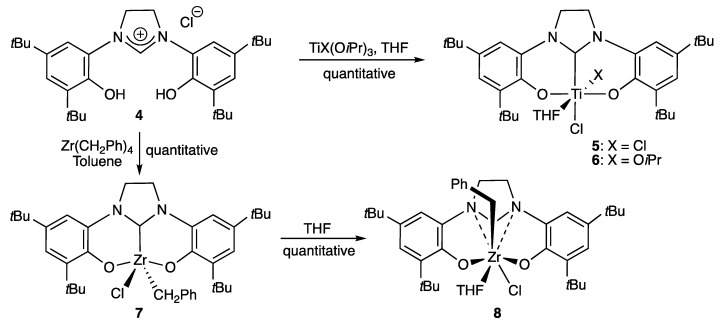
Preparation of Ti(IV) and Zr(IV) pincer complexes **5**–**7** featuring a more rigid bis(aryloxide) NHC ligand from the imidazolium salt **4**, and unexpected rearrangement to the benzyl complex **8** [51,52,55].

More rigid representatives of the same family were prepared via a different route, which consists of the elimination of alcohols from imidazolium salts. For instance, Ti(IV) complexes **5** and **6** were quantitatively formed by the reaction of the imidazolium salt **4** with TiCl(O*i*Pr)_3_ and Ti(O*i*Pr)_4_ precursors, respectively (Scheme 4) [51]. Deduced from a solid state analysis, complexes **5** and **6** exhibit a slightly distorted octahedral geometry with the bis(aryloxide) NHC ligand coordinated in a meridional manner. The NHC Ti(IV) complex **6** was shown to readily initiate the ring-opening polymerization of *rac*-lactide in a controlled fashion without the NHC a priori playing any role in the catalytic transformation. Related pincer complexes were obtained using the same protocol in the Zr(IV) and Hf(IV) series [52]. Despite lower yields, amine elimination was also found to be a possible route to afford such pincer complexes. The reaction of salt **4** with M(NMe_2_)_4_ (M = Ti, Zr) thus afforded the corresponding bis(phenoxy) NHC amido complexes.

Homoleptic bis-ligated NHC complexes could be also synthetized in high yields by reacting the imidazolium salt **4** with half of an equivalent of MCl_4_ complexes (M = Ti, Zr, Hf) in the presence of Et_3_N [53]. In all complexes, the metal atom adopts a distorted octahedral geometry with the two NHCs located in *trans* position one from each other. These three NHC pincer complexes were described to be redox-active and, in the case of Zr and Hf derivatives, to present luminescent properties, constituting the first examples of emissive non-metallocene group 4 metal complexes. It must be mentioned that tetravalent Ti(IV) complexes bearing the same pincer ligand were found to be active and selective for the copolymerization of cyclohexene oxide with CO_2_ [54].

In the Zr series, toluene elimination was observed from the imidazolium salt **4** by using Zr(CH_2_Ph)_4_ as a metal precursor leading to the corresponding chlorobenzyl NHC pincer complex **7** in quantitative yield (Scheme 4) [55]. However, while this complex is stable in non-coordinating solvents, in the presence of THF the quantitative formation of the unexpected Zr-THF adduct **8** as a single isomer was observed. The complex **8** is characterized by the presence of a heptacoordinate Zr center featuring an *O,N,C,N,O*- pentadentate trianionic ligand. The formation of **8** was rationalized through a Lewis base assisted benzyl migration from the Zr atom to the carbenic center. This unusual benzyl migration was also observed with other group 4 metals (Ti, Hf) [56]. More recently, the reversibility of this benzyl migration was demonstrated by the addition of PMe_3_ to a bis(phenolate) benzylimidazolylidene(dibenzyl)zirconium complex and then abstraction of the coordinated PMe_3_ on the Zr complex formed with a Ni(0) complex [57].

**Scheme 5:**
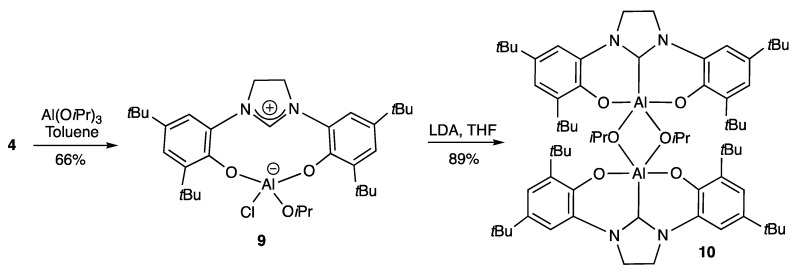
Preparation of the dimeric Al(III) complex **10** featuring a more rigid bis(aryloxide) NHC ligand from the imidazolium salt **4** [64].

Bis(aryloxide) NHC pincer ligands were successfully coordinated to a wide variety of metal centers, such as metals of groups 5 (V) [58], 6 (Mo) [59], 7 (Mn) [58], 9 (Ir [60], Co [61,62]), 10 (Ni, Pd, Pt) [63], and 13 (Al) [64]. More precisely from the imidazolium salt **4**, high oxidation sate V(V) and Mn(III) metal complexes were readily prepared using triisopropoxyvanadium(V) oxide [(*i*PrO)_3_V=O] and manganese(III) acetylacetonate [Mn(aca)_3_] precursors, respectively [58]. Al(III) complexes supported by such a dianionic NHC pincer ligand were obtained via various synthetic methods [64]. A possible route involved first, via alcohol elimination, the formation of the zwitterionic bis(phenolate) imidazolinium complex **9** featuring a four-coordinate tetrahedral Al(III) center *O,O*- chelated by the two phenolate extremities (Scheme 5). The latter was then readily converted to the pincer NHC Al(III) complex **10** upon reaction with an equivalent of LDA. The dimeric nature of **10** is indicated by an X-ray diffraction analysis where the two Al centers are supported by a *O,C,O*- coordinated NHC bis(phenolate) ligand and connected to one another via *μ*-O bridging Al-O*i*Pr groups. Both Al centers display a slightly distorted trigonal bipyramidal geometry with a meridional coordination of the bis(phenolate) NHC ligand. Moreover, the pincer Al complex **10** was reported to efficiently polymerize *rac*-lactive and trimethylene carbonate in a highly controlled fashion for the production of narrow disperse materials with catalytic performance in the range of conventional group 13 based ROP catalysts.

**Scheme 6:**
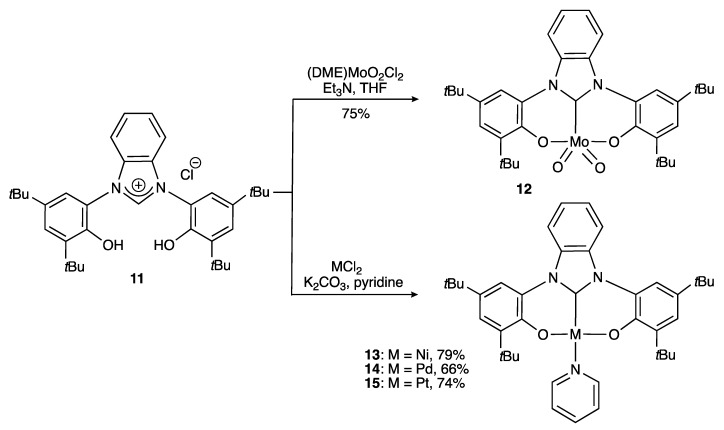
Preparation of metal complexes **12**–**15** (M = Mo, Ni, Pd, Pt) from the benzimidazolium salt **11** [59,63].

To accommodate heavy metal centers, the introduction of a benzimidazol-2-ylidene core was also considered, on the assumption that the presence of a π-conjugated system over the three six-membered aromatic rings should provide additional stabilization to the metal center. High-valent NHC Mo(VI) complexes featuring a *O,C,O*- benzimidazolylidene pincer ligand were prepared, as illustrated with the complex **12** isolated in 75% yield by addition of (DME)MoO_2_Cl_2_ in THF to the benzimidazolium precursor **11** in the presence of Et_3_N (Scheme 6, top) [59]. In the solid state, the dioxo complex **12** was found to crystallize following two different arrangements, namely a dimeric structure with two six-coordinate Mo(VI) atoms in a strongly distorted octahedral environment and a monomeric form with the five-coordinate Mo(VI) center lying in between a square pyramidal and a trigonal bipyramidal geometry. The dioxo complex **12**, which represents a rare example of five-coordinated Mo(VI) complex, was found to be stable toward air and moisture in the solid state and in solution. The pre-ligand **11** was also reported to stabilize oxo-imido and bis(imido) Mo(VI) pincer complexes.

A series of group 10 metal complexes, **13**–**15**, was prepared in good yield (66–79%) when the same precursor **11** was treated with one equivalent of MCl_2_ (M = Ni, Pd, Pt) in the presence of an excess of potassium carbonate in pyridine at 100 °C (Scheme 6, bottom) [63]. This one-pot procedure was also successfully applied in the imidazolylidene series. Imposed by the tridentate NHC, these complexes adopt a distorted square-planar geometry. DFT studies performed on Pd and Pt representatives indicated that the HOMO is centered on the metal and the aryloxide fragment while the LUMO involves mainly the pyridine co-ligand. Based on these findings, optical properties were studied evidencing the absence of luminescence at room temperature but in the case of Pt complexes the presence of an emission band in the green region of the visible spectrum at 77 K, which was attributed to a long-lived triplet-manifold excited state featuring MLLCT character [63].

**Scheme 7:**
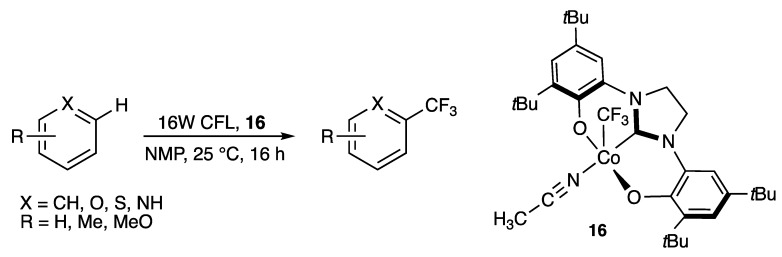
Photoinduced trifluoromethylation of (hetero)arenes by the pincer Co(III) complex **16** [62].

In the Co series, the trifluoromethylation of (hetero)arenes was reported, thanks to the thermally stable pincer Co(III) complex **16**, which is able under light exposure to release a CF_3_ radical (Scheme 7) [62]. This result represents an elegant strategy to activate strong M-CF_3_ bonds for the functionalization of small molecules. From a synthetic point of view, the Co(III) complex **16** was formed by oxidation of the corresponding four-coordinate Co(II) complex using AgCF_3_ in MeCN. An X-ray diffraction analysis of **16** indicates that the CF_3_ group is orthogonal to the *O,C,O*- Co plane with a short Co-CF_3_ bond in favor of a high degree of covalency. The complex **16** appeared to be diamagnetic well described as a low-spin Co(III) center stabilized by a closed-shell dianionic bis(phenolate) NHC core pincer ligand.

**Scheme 8:**
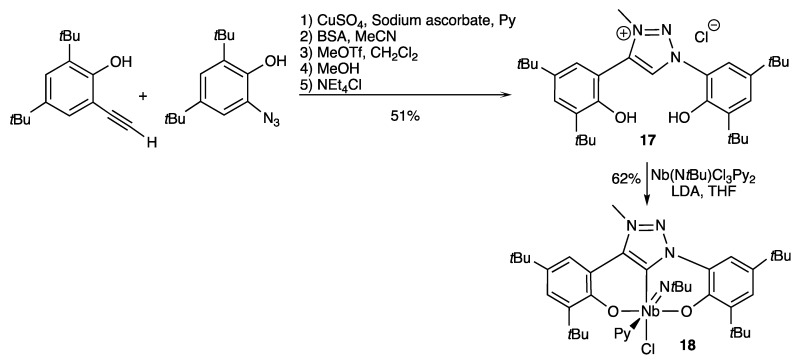
Preparation of the Nb complex **18** featuring a bis(phenolate) mesoionic carbene ligand [65].

Very recently, the first bis(phenolate) mesoionic carbene was reported by Hohloch et al. following a ten-step procedure and was shown to be a valuable ligand for the preparation of various heteroleptic early transition metal pincer complexes (group 4 to group 6) (Scheme 8) [65]. The triazolium salt **17**, which can be prepared on a multi-gram scale, was obtained by reacting phenyl substrates bearing alkyne and azide functionalities under classical click conditions. Prior to N-methylation, the two alcohol functions were protected in the presence of bis(trimethylsilyl)acetamide (BSA). Treatment with MeOH followed by salt metathesis with tetraethylammonium chloride afforded finally the pre-ligand **17** in 51% overall yield. From the latter, rare examples of Ti, Mo, and Nb imido complexes were prepared, as illustrated with the Nb complex **18** isolated in 62% yield. The Nb atom in **18** resides in a distorted octahedral environment where the coordination sphere is completed by the *O,C,O*- pincer ligand and imido, chloro, and pyridine co-ligands. Comparison of the electrochemical properties of these carbenic complexes with more classical bis(phenolate) NHC complexes conclude that, as expected, triazolylidenes are more difficult to oxidize than imidazolinylidene-based systems.

**Scheme 9:**
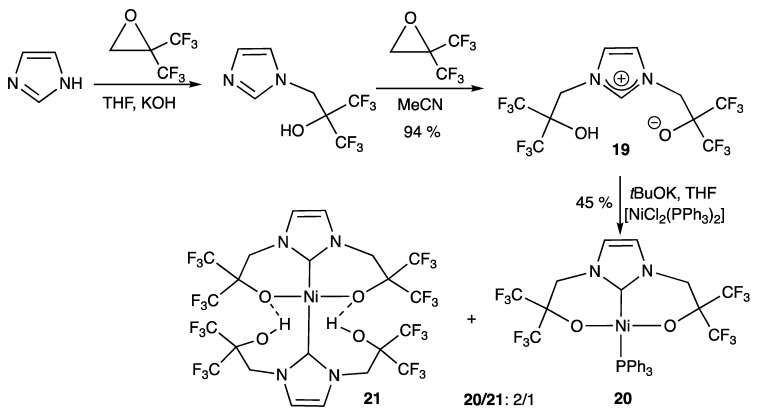
Preparation of bis(alkoxy) NHC Ni(II) complexes **20** and **21** from 1*H*-imidazole [69].

After the development of various bis(aryloxy) NHC core systems, bis(alkoxy) analogues were naturally considered. For such purpose and based on preliminary studies on bidentate systems [66,67], the introduction of electron-withdrawing CF_3_ groups was envisaged to decrease the acidity of the alcohol hydrogen atoms. Indeed, the stability of fluoroalkoxy carbenes has been reported, in particular the reluctance of the alkoxy fragment not to react with the electrophilic imidazolium center as may be the case with more basic alkoxides [68]. Under phase-transfer catalysis, the imidazolium salt **19** as a stable zwitterion was thus prepared through a sequential method by reacting 1*H*-imidazole with hexafluoroisobutylene oxide (Scheme 9) [69]. It is noteworthy that the salt **19** could be obtained in excellent yield (94%) via a one-pot procedure without isolating the imidazole intermediate. This methodology was extended to 3,4-disubstituted imidazole and triazole derivatives. Treatment of **19** with two equivalents of *t*BuOK generated the corresponding free carbene, which was observed to be relatively stable in solution. Addition of [NiCl_2_(PPh_3_)_2_] to this carbene resulted in the formation of the NHC pincer **20** and the bis(NHC) complex **21** in a 2/1 ratio. While an X-ray diffraction analysis of **20** confirmed the square-planar geometry around the Ni(II) center with the meridional coordination of the bis(alkoxy) NHC that of **21** indicated that two alkoxy groups of the same NHC are bound to Ni and the two hydroxyl groups from the other NHC are hydrogen-bonded to the adjacent oxygen atoms.

#### 2.1.2. With Two Coordinating S- (or Se) Atoms

**Scheme 10:**
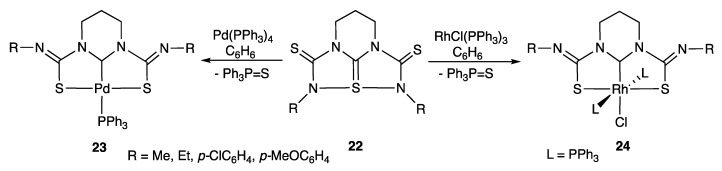
Preparation of tridentate bis(thiolate) NHC Pd(II) and Rh(III) complexes **23** and **24** from tetraazapentalene derivatives **22** [70].

Bis(thiolate) NHC core complexes were actually reported before their oxygenated analogues. The first complexes were obtained by reacting Pd(PPh_3_)_4_ and RhCl(PPh_3_)_3_ with tetraazapentalene derivatives **22**, the latter exhibiting unique reactivity due to the presence of hypervalent sulfur (Scheme 10) [70]. In both cases, the formation of Pd(II) and Rh(III) complexes **23** and **24** was accompanied by the release of triphenylphosphine sulfide. It is noteworthy that, depending on the nature of the amine substituents (R), the bis(thiolate) NHC complexes were isolated as a mixture with their isostructural complexes containing an unsymmetrical amido, NHC, thiolate pincer ligand, knowing that more hindered R substituents, disadvantage a priori the latter form. The best ratios in favor of the *S,C,S*- pincer complexes **23** and **24** were observed with small *p*-Cl- and *p*-MeOC_6_H_4_ amine substituents with yields between 86% and 99%. The complexes based on a *N,C,S*- ligand were generally found to be less stable than their analogues exhibiting the symmetrical *S,C,S*- ligand. In a Pd complex of type **23** bearing N-Me groups, an X-ray diffraction analysis was performed confirming the square planar geometry around the Pd(II) center surrounded by two sulfur, one phosphorus, and a carbenic carbon atom. This Pd complex was found to be very stable in organic solvents under air. For Rh(III) complexes of type **24**, the octahedral geometry of the metal center with two phosphine ligands located in *trans* position was deduced on the basis of a solid state analysis performed in the case of a Rh complex featuring the hybrid *SCN*- pincer ligand. Following the same strategy, a diselenato version of **22** based on a five-membered saturated NHC was prepared by the same authors and coordinated with different metal centers such as Pd(PPh_3_)_4_, Pt(PPh_3_)_4_, and RhCl(PPh_3_)_3_, affording corresponding *Se,C,Se*- pincer complexes. [71,72]

**Scheme 11:**
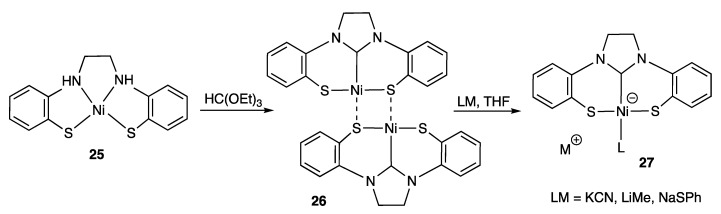
Preparation of bis(thiolate) NHC Ni(II) pincer complexes **27** through dissociation of the dimeric precursor **26** [73].

Another representative of the family was described by Sellmann et al. who observed the unexpected formation of bis(thiolate) NHC Ni(II) pincer complexes **27** upon dissociation of the dimeric complex **26** by addition of different metallic salts (KCN, LiMe, NaSPh) (Scheme 11) [73]. X-ray structure determinations confirmed the square planar geometry around the metal center with a characteristic propeller-like twist resulting from positioning the phenyl rings above and below the coordination plane. If the pincer complexes **27** exhibit remarkable thermal stability, in the presence of Brönsted acids, the regeneration of dimer **26** was noticed.

#### 2.1.3. With Two Coordinating N- Atoms

**Scheme 12:**
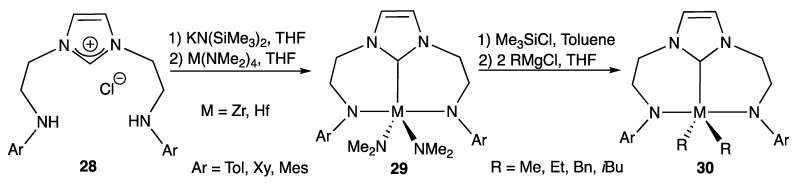
Preparation of bis(amido) NHC pincer complexes (M = Zr, Hf) **29** and **30** from the imidazolium salt **28** [74,75].

The first bis(amido) NHC pincer complex was described by Fryzuk et al. in 2004 in the Zr(IV) series [74]. The corresponding pre-ligand, namely the bis(amine) imidazolium salt **28** (Ar = Tol) was synthetized by the reduction of a bis(amide) imidazolium salt using borane-dimethylsulfide. Later, in order to introduce larger N-aryl groups (Ar = Mes, Xy), an alternative route was developed consisting of melting the appropriate N-substituted imidazole with a *β*-chloroethylarylamine [75]. Whatever the preparation method, imidazolium salts **28** were treated with KN(SiMe_3_)_2_ to give in good yield the stable free NHCs substituted by two amine donor arms. The latter reacted cleanly with M(NMe_2_)_4_ complexes (M = Zr, Hf), providing a convenient entry to *N,C,N*- group 4 pincer complexes (Scheme 12). The dimethylamido co-ligands could be readily removed by adding an excess of chlorotrimethylsilane, affording the corresponding dichloride adducts which were then converted into dialkyl complexes by treatment with Grignard reagents. In the case of a Hf complex of type **30** bearing two isobutyl groups, an X-ray diffraction analysis evidences a distorted trigonal bipyramidal geometry around the metal atom with the two amido moieties in pseudo-*trans* position [75]. It should be noted that Hf dialkyl complexes are more thermally stable than Zr representatives, with the exception of the Hf diethyl complex which undergoes *β*-hydrogen transfer and subsequent C-H bond activation with the neighboring N-Mes substituent to afford a cyclometalled complex [75]. The Hf dialkyl complexes were also shown to insert carbon monoxide and isocyanides to give the related η^2^- acyl and η^2^- iminoacyl derivatives. After activation with [Ph_3_C, B(C_6_F_5_)_4_], the Zr dimethyl complex showed moderate catalytic activity for ethylene polymerization.

**Scheme 13:**
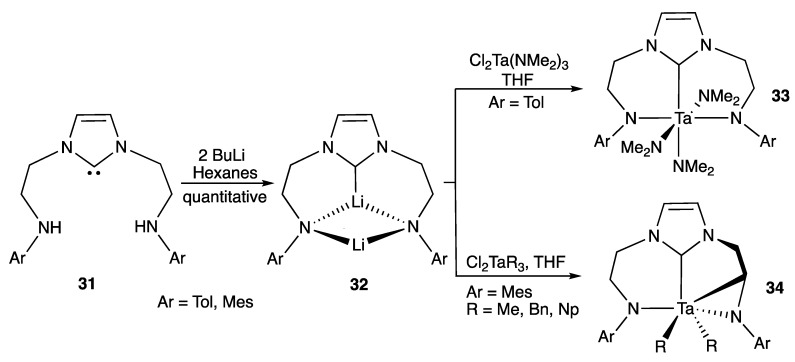
Preparation of bis(amido) NHC Ta(V) complexes **33** and **34** from the free NHC **31** [76].

From the same pre-ligand, bis(amido) NHC tantalum pincer complexes were also reported [76]. However, to reach such systems, the formation of the dilithiated diamido NHC ligand **32** prepared by addition of 2 equivalents of BuLi to the free carbene **31** was a prerequisite. Indeed, from the free carbene **31**, only the formation of bidentate amido NHC Ta complexes were observed, the remaining amine arm being reluctant to coordinate the metal center. By contrast, the metathesis reaction of **32** in the presence of various TaCl_x_(NMe_2_)_5−x_ precursors proceeded under mild conditions and in good yield to afford the desired pincer complexes, as illustrated with complex **33** (Scheme 13, top). However, attempts to synthetize trialkyl derivatives following this methodology were not successful, yielding instead metallaziridines, as demonstrated with the formation of *N,C,C,N*- NHC Ta(V) complexes **34** (Scheme 13, bottom). An X-ray diffraction analysis of one of these representatives (with R = Np) emphasizes the facial orientation of the activated C-H bond and the distorted pseudo trigonal bipyramidal geometry around the Ta center. DFT calculations confirm that such cyclometallated species formed by the endocyclic C-H activation of one of the amido arms are thermodynamically favored over the targeted trialkyl derivatives.

**Scheme 14:**
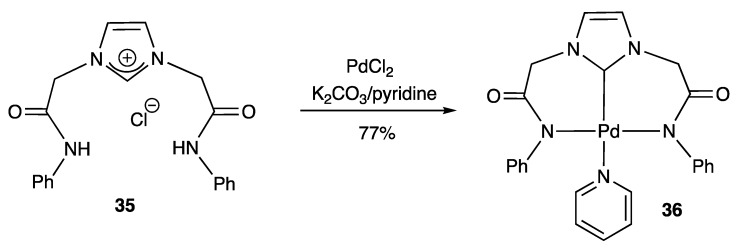
Preparation of the bis(amido) NHC Pd(II) pincer complex **36** from the imidazolium salt **35** [77].

Bis(amido) NHC ligands were also coordinated to group 10 metal centers. The Pd(II) complex **36** was prepared from the tridentate imidazolium salt **35** exhibiting two side amide arms and PdCl_2_ in the presence of the K_2_CO_3_/pyridine system (Scheme 14) [77]. This square planar pincer Pd complex isolated in 77% yield showed significant catalytic activity in Suzuki coupling reactions of aryl bromides with phenylboronic acid, although less than corresponding monodentate NHC Pd complexes bearing a pendant neutral amine arm. This difference was attributed to the too strongly anionic amido coordinating ends, which do not allow in the case of **36** the release of vacant sites during the catalytic process. The dissociation of the neutral NHC moiety is expected to be more favorable with electropositive early transition metal centers.

#### 2.1.4. With Two Coordinating C- Atoms

Phosphonium ylides are globally neutral in their free state, characterized by an almost planar carbanion stabilized by an adjacent tetrahedral phosphonium center. In their coordination state, phosphonium ylides act exclusively as η^1^- carbon centered ligands, rather than as η^2^- C=P ligands [78], and can therefore be considered locally as anionic carbon ligands [79]. This chemical description means that phosphonium ylides like NHCs behave as strong σ-donor carbon ligands while differing in their bonding mode (NHC, 2e^−^ donor (L type); P^+^-ylide, 1e^−^ donor (X type)) [80,81]. Following preliminary reports in the field aimed at developing ylide-based metal complexes [82,83,84,85], NHC and phosphonium ylide donor moieties were recently associated by a C_3_-propyl bridge in the bi- [86], tetradente [87], and in the pincer series forming very electron-rich complexes [88], thanks to the design of a general synthetic strategy [89].

**Scheme 15:**
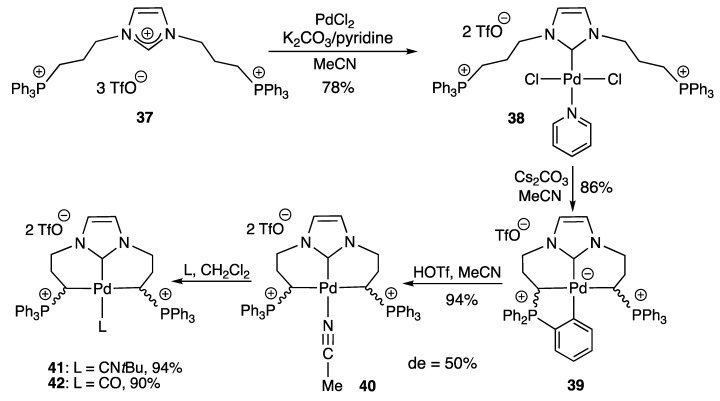
Preparation of NHC, diphosphonium bis(ylide) Pd(II) pincer complexes **40**–**42** from the imidazolium salt **37** [88].

A new family of pincer Pd(II) complexes bearing an electron-rich *C,C,C*- NHC, diphosphonium bis(ylide) ligand was indeed prepared from the imidazolium salt **37** featuring two phosphonium side chains obtained through the dual N-functionalization of 1*H*-imidazole by (3-bromopropyl)triphenylphosphonium bromide (Scheme 15) [88]. Due to a difference in acidity between the cationic moieties, the imidazolium salt **37** was sequentially coordinated to Pd(II) centers leading first to the NHC Pd complex **38** and then to the *ortho*-metallated Pd complex **39**. Protonation of the latter afforded the NHC, diphosphonium bis(ylide) pincer Pd(II) complex **40** as a mixture of *meso*- and *dl*-diastereomers (de = 50%). The pincer complex **40** isolated in 94% yield appeared to be perfectly stable in air both in the solid state and in solution. The selectivity of C-coordination was rationalized on the basis of DFT calculations, indicating the quasi-degeneracy of the two diastereomeric forms. Thanks to its electronic properties, this NHC core pincer was shown to efficiently stabilize Pd(II) complexes bearing isocyanide **41** and carbonyl co-ligands **42**, whose examples of this latter type remain rare due to easy Pd–CO dissociation (Scheme 15). In the isocyanide case, an X-ray diffraction analysis of both isomers was achieved showing that the Pd(II) atom is an integral part of two strongly distorted fused six-membered metallacycles and resides in a quasi-square planar environment with the two phosphonium ylides occupying mutually *trans* positions [88].

NHC core diphosphonium bis(ylide) Pd complexes **40**–**42** represent the sole examples of LX_2_-type pincer complexes where the metal center is bonded only with carbon atoms, in the present case being of different nature: one carbenic (*sp*^2^) and two ylidic (*sp*^3^) carbon atoms. Their availability combined with their stability should benefit catalytic processes requiring extremely electron-rich ligands.

### 2.2. Based on Two Different Side Arms

The interest in unsymmetrical pincer ligands has considerably increased in recent years since they can provide significantly different chemical donor extremities with a more or less pronounced hard/soft character, thus affording metal complexes with unprecedented properties. This may lead in particular to catalysts exhibiting unique reactivity and selectivity profiles. In this direction, NHC core pincers of LX_2_-type bearing two different side arms are rare and their synthesis represent an additional synthetic challenge because their formation requires the coordination of three different donors having their own chemical features at the same metallic center. For instance, the coordination of neutral and/or anionic donor ends possessing a wide range of basicity will have to take into account the relative acidity of each H-atom in the corresponding conjugated acid precursors.

#### 2.2.1. With N,O- Coordinating Atoms

**Scheme 16:**
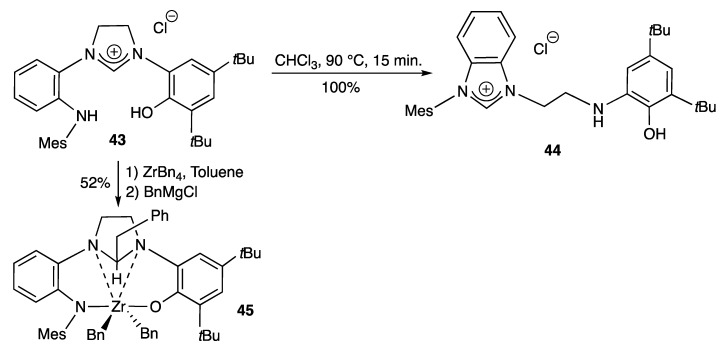
Preparation of the tridentate *N,C,O*- imidazolium salt **43** with corresponding thermal rearrangement to the benzimidazolium salt **44** and coordination to the Zr(IV) complex **45** [90,91].

In the last decade, the preparation of the imidazolium salt **43** bearing pending amine and phenol arms was reported in four steps in 52% overall yield from 2-(*N*-mesitylamino) aniline [90]. However, while this cation appeared to be stable in the solid state, it was observed to quantitatively rearrange in solution to form the thermodynamically favored benzimidazolium salt **44** (Scheme 16) [91]. DFT calculations were performed to rationalize the mechanism of this unprecedented rearrangement. Despite its unstability, various attempts to coordinate the pre-ligand **43** were carried out with group 4 metals, all leading to unexpected species but not to the desired pincer complexes. It is worth mentioning the formation of the Zr(IV) complex **45**, which was obtained when the precursor **43** was reacted with ZrBn_4_ and BnMgCl in toluene. The formation of **45** results formally from the migration of a benzyl group and a proton to the carbenic center, converting the heterocycle to an imidazolidine. The latter stands as a Zr(IV) complex where the metallic center interacts with a tetradentate *N,N,N,O*- dianionic ligand featuring two X-type (amide and phenoxide) and two L-type (amine) donor moieties. Similar benzyl migration was already evidenced in Zr(IV) complexes supported by bis(phenoxy) NHC pincer ligands [55]. These surprising results tend to illustrate the difficulty of access to unsymmetrical pincer systems containing different coordinating ends.

#### 2.2.2. With N,S- Coordinating Atoms

**Scheme 17:**
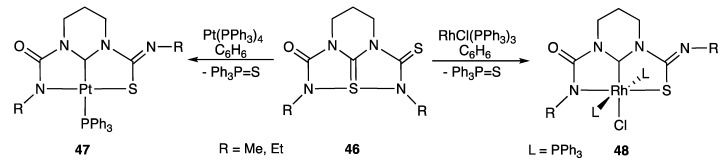
Preparation of *S,C,N*- pincer complexes **47** (M = Pt) and **48** (M = Rh) from unsymmetrical tetraazapentalenes **46** [92].

The method developed to prepare *S,C,S*- pincer complexes (see Scheme 10) also enabled the synthesis of unsymmetrical pincer complexes as illustrated with the formation of the *S,C,N*- Pt(II) **47** and Rh(III) **48** complexes. The difference lies in the nature of the tetraazapentalene substrates **46** where a thiocarbonyl function is replaced by a carbonyl group (Scheme 17) [92]. As demonstrated in the Rh(III) series, the substitution of both thiocarbonyls by two carbonyl groups does not lead, as one might have expected, to the formation of *N,C,N*- pincers but to a bidentate *N,S*- Rh complex [92].

#### 2.2.3. With C,O- Coordinating Atoms

**Scheme 18:**
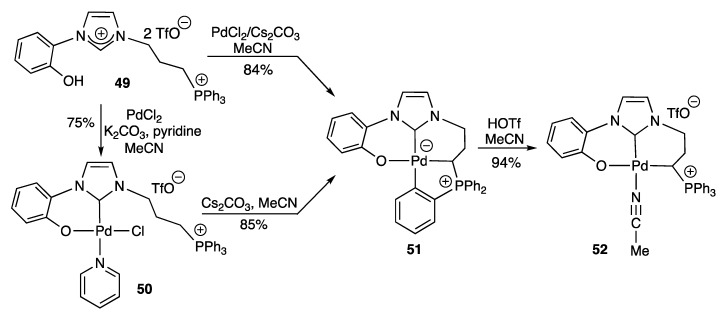
Preparation of the *C,C,O*- phenolate, NHC, phosphonium ylide pincer Pd(II) complex **52** from the imidazolium salt **49** following two different routes [93].

With the previous cases **47**–**48**, the Pd(II) complex **52** constitutes one of the very rare examples of NHC core pincer complex of LX_2_-type exhibiting two different peripheral coordinating extremities reported to date [93]. The latter, featuring pending phenolate and phosphonium ylide moieties, was readily obtained from the *ortho*-metallated Pd complex **51** in 94% yield, thanks to the selective acid cleavage of a C_ar_-Pd bond using HOTf in MeCN (Scheme 18). The highly strained zwitterionic *C,C,C,O*- Pd complex **51** was prepared through two distinct routes, either directly from the tridentate imidazolium salt **49** by adding PdCl_2_ in the presence of Cs_2_CO_3_ in 84% yield or in a two-step procedure via the NHC Pd pyridine adduct **50** in 64% overall yield. The pincer complex **52** was readily converted to its isocyanide analogue **53** by an exchange reaction at the Pd center. In this case, the formation of a Pd–CO adduct was not experimentally observed, due probably to the too weak donor character of the *C,C,O*- pincer ligand.

**Scheme 19:**
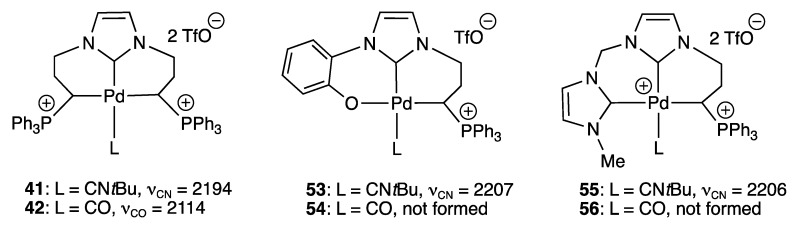
Experimental IR ν_CO_ and ν_CN_ stretching frequencies (cm^−1^) for NHC core phosphonium ylide-based pincer Pd(II) complexes (L = CN*t*Bu or CO) [93].

The overall donating character of the *C,C,O*- ligand of complex **52** was further analyzed on the basis of IR ν_CO_ and ν_CN_ stretching frequencies, oxidation potentials, and DFT calculations by comparison with isostructural phosphonium ylide-based pincer Pd complexes. Notably, the IR ν_CN_ frequency values of Pd complexes **53** (2207 cm^−1^) and **55** (2206 cm^−1^) indicate that the *C,C,O*- NHC, phenolate, phosphonium ylide, and the *C,C,C*- bis(NHC) phosphonium ylide have similar electronic properties. These IR values, which appear at higher frequency than that of the Pd-CN*t*Bu complex **41** (2194 cm^−1^), bearing the *C,C,C*- bis(ylide) NHC ligand lead to the conclusion that the substitution of a NHC or a phenolate for a phosphonium ylide increases significantly the donor character of corresponding pincer ligands (Scheme 19) [93]. The Pd−CO complex **42** only experimentally observed in the case of the bis(ylide) ligand is in perfect agreement with these findings. This trend was also found to be in line with previous studies performed on an isoelectronic series of *C,C*- chelating NHC, phosphonium ylide Rh(CO)_2_ complexes. [94,95]

The electronic properties of these phosphonium ylide-based pincer Pd(II) complexes were exploited in homogeneous catalysis for the Pd-catalyzed allylation of aldehydes. It was in particular observed that the Pd complex bearing the most donor pincer ligand, namely the NHC, bis(ylide), was the most active in this catalytic process [93].

## 3. Conclusions and Perspectives

In perpetual search for new ligands, those based on pincer architecture have a promising future, not only because of a structural diversity, which remains to be discovered, but also because of their many potential applications, especially in the fields of organometallic chemistry, homogeneous catalysis, and materials. In this large family, pincer ligands of LX_2_-type which position two anionic donor units on either side of a neutral central donor are less common and undoubtedly deserve to be more considered. Combining neutral with anionic donor extremities indeed offers several advantages such as the efficient coordination of a wide range of metal centers and the access to unusual oxidation states. For instance, anionic donors are more likely to bind early transition metals, while neutral ones generally prefer to coordinate late transition metals. In this category of pincer ligands, NHCs substituted by two anionic arms represent a promising family. The association of NHCs with anionic donors indeed makes it possible to stabilize but also to modulate the reactivity of various metal complexes across the periodic table. Independently of the two anionic arms, the nature of the central donor allows a fine adjustment of the electronic properties of the pincer structure since saturated, unsaturated, five- or six-membered NHCs, as well as mesoionic carbenes can be introduced. However, the preparation of such chelating systems generally represents a synthetic challenge in terms of coordination selectivity and choice of metal center in order to reach a good compromise between stability and reactivity. As pointed out in this review, the nature of the donors used in this family of pincer ligand remains very limited to date, and there is no doubt that the introduction of other donor functionalities should allow the development of new properties in the area of redox and photo-active systems but would also be beneficial for the activation of small molecules and for homogeneous catalysis.
